# Risk of contracting pneumonia among patients with predialysis chronic kidney disease: a population-based cohort study in Taiwan

**DOI:** 10.1051/bmdcn/2017070320

**Published:** 2017-08-25

**Authors:** Shih-Wei Lai, Cheng-Li Lin, Kuan-Fu Liao

**Affiliations:** 1 College of Medicine, China Medical University Taichung 404 Taiwan; 2 Department of Family Medicine, China Medical University Hospital Taichung 404 Taiwan; 3 Management Office for Health Data, China Medical University Hospital Taichung 404 Taiwan; 4 Graduate Institute of Integrated Medicine, China Medical University Taichung 404 Taiwan; 5 College of Medicine, Tzu Chi University Hualien 970 Taiwan; 6 Department of Internal Medicine, Taichung Tzu Chi General Hospital Taichung 427 Taiwan

**Keywords:** Pneumonia, Predialysis chronic kidney disease, Taiwan National Health Insurance Program

## Abstract

Objectives: The objective of the study was to investigate the association between predialysis chronic kidney disease and contracting pneumonia in Taiwan.

Methods: We employed a population-based, retrospective cohort design using the database of the Taiwan National Health Insurance (NHI) Program. There were 18807 subjects aged 20-84 years who were newly diagnosed with predialysis chronic kidney disease between 2000 to 2012 as the predialysis chronic kidney disease group and 18807 randomly selected subjects without chronic kidney disease as the non-chronic kidney disease group. The predialysis chronic kidney disease and non-chronic kidney disease groups were matched according to sex, age, comorbidities, and the year of index date. The incidence of contracting pneumonia among both groups at the end of 2013 was calculated. The multivariable Cox proportional hazards regression model was used to calculate the hazard ratio (HR) and 95% confidence interval (CI) for contracting pneumonia being associated with predialysis chronic kidney disease.

Results: The overall incidence of contracting pneumonia was 1.47-fold higher in the predialysis chronic kidney disease group than that in the non-chronic kidney disease group (24.6 vs. 16.7 per 1, 000 person-years, 95% CI 1.40, 1.55). After adjusting for co-variables, the HR of contracting pneumonia became 1.52 for subjects with predialysis chronic kidney disease (95% CI 1.43, 1.60) compared to subjects without chronic kidney disease. With even further analysis, in the absence of any comorbidity, the adjusted HR of contracting pneumonia was 1.53 for subjects with predialysis chronic kidney disease alone (95% CI 1.32, 1.76).

Conclusions: Patients with predialysis chronic kidney disease have a 1.52-fold increased risk of contracting pneumonia as compared to those with non-chronic kidney disease. Even in the absence of any comorbidity, a greater than average risk of contracting pneumonia remains present.

## 1. Introduction

Pneumonia continues to be a major public health problem due to the substantial number of deaths it causes as well as the high economic cost to treat it globally. Pneumonia has been ranked as the third leading cause of death in the world.[1] In addition, pneumonia was the fourth most common cause of death in Taiwan in 2015.[2] Lin *et al*’s study showed that patients with pneumonia needed longer lengths of hospital stays than average to recover and higher health care expenditures, both of which have placed a significant economic burden on Taiwan. [3]

Meanwhile, chronic kidney disease was the ninth most common cause of death in Taiwan in 2015.[2] Much evidence has already shown that pneumonia is a common comorbidity among patients with chronic kidney disease.[4, 5] Thus, we made a rational hypothesis that there could be a potential link between predialysis chronic kidney disease and pneumonia in Taiwan. If the link can be made, from a view of preventive medicine, vaccination for pneumonia might be something to take into account among patients with predialysis chronic kidney disease. With the above in mind, our study employed apopulation-based cohort design using the database of the Taiwan National Health Insurance (NHI) Program to investigate the association between predialysis chronic kidney disease and pneumonia.

## 2. Methods

### 2.1. Study design and data source

For this study, a population-based, retrospective cohort design was employed using the database from the Taiwan National Health Insurance Program. Taiwan is an independent country with more than 23 million persons. The NHI Program was launched in March 1, 1995, and it now covers about 99% of the said 23 million persons living in Taiwan.[6] The details of the insurance program will not be discussed here, but they have been thoroughly written about in previous studies.[7–11] Our study was approved by the Research Ethics Committee of China Medical University and Hospital in Taiwan (CMUH-104-REC2-115).

### 2.2. Selection of subjects

Subjects aged 20-84 who had been newly diagnosed with predialysis chronic kidney disease (International Classification of Diseases, Ninth Revision, Clinical Modification, ICD-9 codes 581-583, 585-587, and 588.8-588.9) between 2000 to 2012 were assigned as our predialysis chronic kidney disease group. The date for subjects being diagnosed with predialysis chronic kidney disease was defined as the index date. For each subject with predialysis chronic kidney disease, one subject without chronic kidney disease was randomly selected from the same database and was assigned to the non-chronic kidney disease group. The predialysis chronic kidney disease group and the non-chronic kidney disease group were matched in terms of sex, age (every 5-year interval), comorbidities, and the year of the former’s index date.

**Fig. 1:**
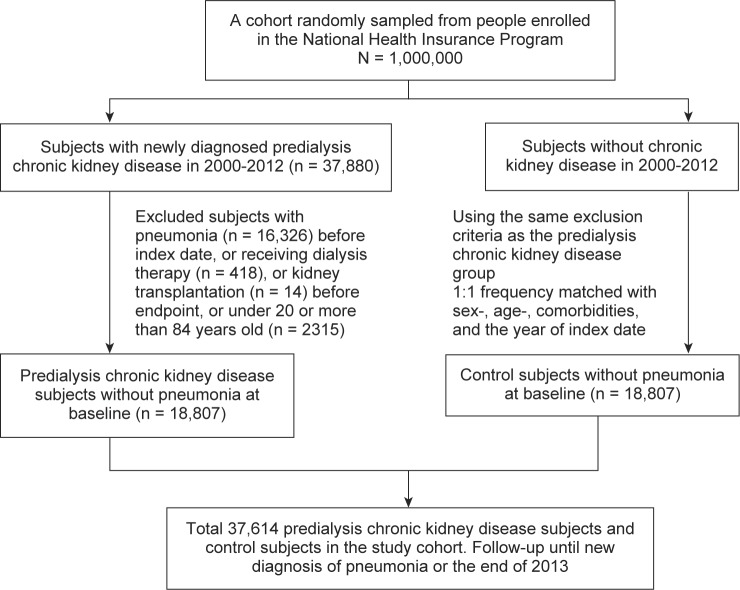
Flow chart for selecting the study’s subjects.

Subjects who had a history of pneumonia (ICD-9 codes 480-486) before the index date were excluded from the study. Subjects who were currently receiving dialysis therapy or had received renal transplantation before the major endpoint were excluded from the study (Fig. 1).

### 2.3. Major endpoint

The major endpoint was a new diagnosis of pneumonia during the follow-up period. Each subject was followed-up with until they were diagnosed with pneumonia, or until the end of 2013.

### 2.4. Comorbid conditions

The comorbid conditions potentially related to contracting pneumonia that were included were as follows: alcohol-related disease, cancer, cardiovascular disease (including coronary artery disease, heart failure, cerebrovascular disease, and peripheral atherosclerosis), chronic liver disease (including cirrhosis, hepatitis B, hepatitis C, and other chronic hepatitis), chronic obstructive pulmonary disease, hyperlipidemia, hypertension, and diabetes mellitus. All comorbidities in the study were diagnosed with ICD-9 codes, which have been well examined in previous studies.[12–21]

### 2.5. Statistical analysis

The differences in the sex, age, and comorbidities between the predialysis chronic kidney disease group and the non-chronic kidney disease group were compared *via* a *Chi*-square test for categorical variables and a *t*-test for continuous variables. The incidence of contracting pneumonia was calculated as the number of pneumonia cases identified during the follow-up period, divided by the total number of follow-up person-years for each group. In the beginning, all variables were included in a univariable model. Then, variables found to be statistically significant in the univariable model were further included in the multivariable model. The multivariable Cox proportional hazards regression model was used to calculate the hazard ratio (HR) with 95% confidence interval (CI) of contracting pneumonia associated with predialysis chronic kidney disease along with other comorbidities. All statistical analyses were performed using SAS 9.2 (SAS Institute, Cary, North Carolina, USA). Two-tailed *P* < 0.05 was considered statistically significant.

## 3. Results

### 3.1. Demorgraphic information and comorbidities of the study population

Table 1 shows the distributions of sex, age, and comorbidities between the predialysis chronic kidney disease and non-chronic kidney disease groups. There were 18807 subjects in the predialysis chronic kidney disease group and likewise 18807 subjects in the non-chronic kidney disease group, with similar distributions of sex and age. The mean ages (standard deviation) of the study’s subjects were 58.5 (15.0) years in the predialysis chronic kidney disease group and 58.2 (15.0) years in the non-chronic kidney disease group (*t*-test, *P* = 0.05). The comorbidities were equally distributed in the predialysis chronic kidney disease group and the non-chronic kidney disease group (*Chi*-square test, *P* > 0.05 for all).

**Table 1:** Demographic information and comorbidities between predialysis chronic kidney disease group and non-chronic kidney disease group.

	Non-chronic kidney disease N=18807		Predialysis chronic kidney disease N=18807		
Variable	n	(%)	n	(%)	*P* value*
Sex					0.99
Female	7655	(40.7)	7655	(40.7)	
Male	11152	(59.3)	11152	(59.3)	
Age group (years)					0.99
20-39	5367	(28.5)	5367	(28.5)	
40-64	6243	(33.2)	6243	(33.2)	
65-84	7197	(38.3)	7197	(38.3)	
Age (years), mean (standard deviation)†	58.2	(15.0)	58.5	(15.0)	0.05
Baseline comorbidities					
Alcohol-related disease	885	(4.71)	885	(4.71)	0.99
Cancer	749	(3.98)	749	(3.98)	0.99
Cardiovascular disease	7604	(40.4)	7604	(40.4)	0.99
Chronic liver disease	4188	(22.3)	4188	(22.3)	0.99
Chronic obstructive pulmonary disease	2468	(13.1)	2468	(13.1)	0.99
Hyperlipidemia	8322	(44.3)	8322	(44.3)	0.99
Hypertension	11699	(62.2)	11699	(62.2)	0.99
Diabetes mellitus	3836	(20.4)	3836	(20.4)	0.99

Data are presented as the number of subjects in each group, with percentages given in parentheses, or the mean with standard deviation given in parentheses.

* *Chi*-square test, and

† *t*-test comparing subjects with predialysis chronic kidney disease and without chronic kidney disease.

### 3.2. Incidence of contracting pneumonia in the study population stratified by sex and age

Table 2 shows that the overall incidence of contracting pneumonia was 1.47-fold higher in the predialysis chronic kidney disease group than that in the non-chronic kidney disease group (24.6 *vs.* 16.7 per 1, 000 person-years, 95% CI 1.40, 1.55). As stratified by sex and age, the incidences of contracting pneumonia were all higher in the predialysis chronic kidney disease group than those in the non-chronic kidney disease group. The incidences of contracting pneumonia increased with age in both groups, with the highest incidence rate being in the predialysis chronic kidney disease group aged 65-84 years (45.2 per 1, 000 person-years).

**Table 2:** Incidence of pneumonia stratified by sex and age between predialysis chronic kidney disease group and non-chronic kidney disease group.

	Non-chronic kidney disease				Predialysis chronic kidney disease					
Variable	N	Event	Person- years	Incidence†	N	Event	Person- years	Incidence†	IRR#	(95% CI)
All	18807	2113	126622	16.7	18807	2821	114713	24.6	1.47	(1.40, 1.55)
Sex										
Female	7655	748	54399	13.8	7655	1035	48472	21.4	1.55	(1.43, 1.68)
Male	11152	1365	72223	18.9	11152	1786	66241	27.0	1.43	(1.34, 1.52)
Age group (years)										
20-39	5367	292	43624	6.69	5367	473	41350	11.4	1.71	(1.53, 1.90)
40-64	6243	569	42385	13.4	6243	794	38994	20.4	1.52	(1.38, 1.66)
65-84	7197	1252	40613	30.8	7197	1554	34369	45.2	1.47	(1.36, 1.58)

† Incidence: per 1, 000 person-years.

# IRR (incidence rate ratio): predialysis chronic kidney disease vs. non-chronic kidney disease (95% confidence interval)

**Fig. 2:**
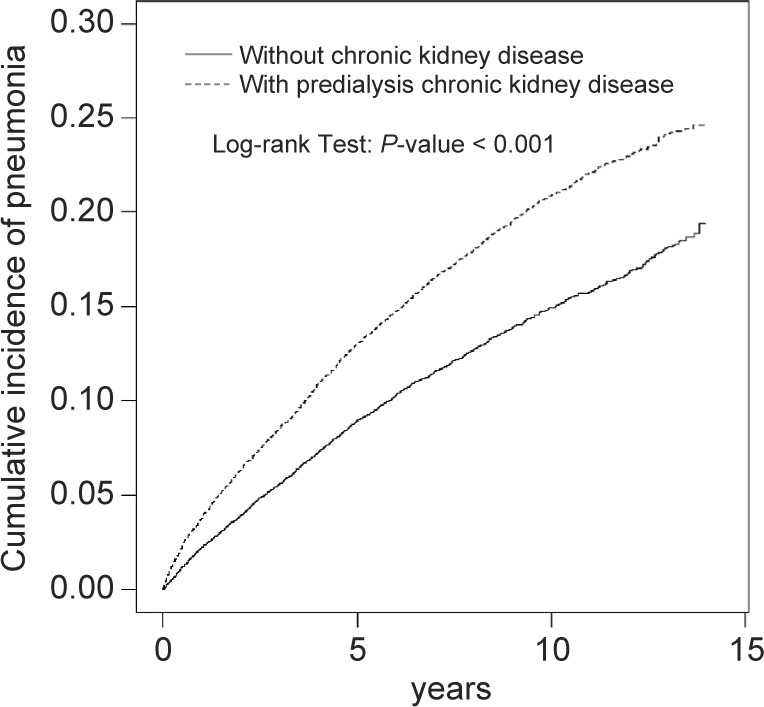
Kaplan–Meier curves showing a significant difference in cumulative incidences of pneumonia among patients with predialysis chronic kidney disease and without chronic kidney disease. (23.0% *vs*. 16.8% at the end of follow-up; *P* < 0.001)

Using the Kaplan-Meier model, we have shown that the cumulative incidence of contracting pneumonia was higher in the predialysis chronic kidney disease group than that in the nonchronic kidney disease group (23.0% vs. 16.8% at the end of follow-up; *P* < 0.001, Fig. 2).

### 3.3. Hazard ratio of contracting pneumonia associated with predialysis chronic kidney disease and comorbidities

Table 3 shows the hazard ratio (HR) of contracting pneumonia associated with predialysis chronic kidney disease and other comorbidities. After adjusting for co-variables, a multivariable Cox proportional hazards regression model showed that the adjusted HR of contracting pneumonia was 1.52 for subjects with predialysis chronic kidney disease (95% CI 1.43, 1.60), compared to subjects without chronic kidney disease. In addition, being male (adjusted HR 1.33, 95% CI 1.25, 1.41), having an alcohol- related disease (adjusted HR 1.35, 95% CI 1.18, 1.55), having cancer (adjusted HR 1.30, 95% CI 1.12, 1.49), having a cardiovascular disease (adjusted HR 1.24, 95% CI 1.17, 1.33), having chronic obstructive pulmonary disease (adjusted HR 1.40, 95% CI 1.31, 1.51), and having diabetes mellitus (adjusted HR 1.59, 95% CI 1.49, 1.69) were other factors associated with contracting pneumonia. Every one-year increase in age was associated with a 1.04-fold increased hazard of contracting pneumonia (95% CI 1.03, 1.04).

**Table 3:** Hazard ratio (HR) and 95% confidence interval of contracting pneumonia associated with predialysis chronic kidney disease and certain comorbidities.

	Crude		Adjusted†	
Variable	HR	(95% CI)	HR	(95% CI)
Sex (male vs. female)	1.29	(1.22, 1.37)	1.33	(1.25, 1.41)
Age (per one year)	1.04	(1.04, 1.05)	1.04	(1.03, 1.04)
Predialysis chronic kidney disease	1.46	(1.38, 1.55)	1.52	(1.43, 1.60)
Baseline comorbidities (yes vs. no)				
Alcohol-related disease	1.27	(1.11, 1.45)	1.35	(1.18, 1.55)
Cancer	1.57	(1.36, 1.80)	1.30	(1.12, 1.49)
Cardiovascular disease	2.15	(2.03, 2.28)	1.24	(1.17, 1.33)
Chronic liver disease	0.93	(0.87, 1.00)	-	-
Chronic obstructive pulmonary disease	2.12	(1.98, 2.26)	1.40	(1.31, 1.51)
Hyperlipidemia	0.96	(0.91, 1.02)	-	-
Hypertension	2.01	(1.89, 2.14)	1.03	(0.96, 1.11)
Diabetes mellitus	1.99	(1.87, 2.11)	1.59	(1.49, 1.69)

† Variables found to be statistically significant in a univariable model were further examined in a multivariable model. Adjustments for sex, age, alcohol-related disease, cancer, cardiovascular disease, chronic obstructive pulmonary disease, hypertension, and diabetes mellitus.

### 3.4. Association of contracting pneumonia stratified by predialysis chronic kidney disease and comorbidities

As a reference of subjects in the study without predialysis chronic kidney disease and without any comorbidity, the adjusted HR of contracting pneumonia was 1.53 for subjects with predialysis chronic kidney disease alone and without any comorbidity (95% CI 1.32, 1.76). The adjusted HR markedly increased to 2.16 for those with predialysis chronic kidney disease and with any comorbidity (95% CI 1.91, 2.43) (Table 4).

**Table 4:** Association of contracting pneumonia stratified by predialysis chronic kidney disease and comorbidities.

Variable		Event	Incidence†	Adjusted HR# (95% CI)
Predialysis chronic kidney disease	Any comorbidity*			
No	No	321	7.82	(Reference)
No	Yes	1792	20.9	1.45 (1.28, 1.64)
Yes	No	468	11.9	1.53 (1.32, 1.76)
Yes	Yes	2353	31.2	2.16 (1.91, 2.43)

† Incidence: per 1, 000 person-years

# Adjusted for sex and age

* Comorbidities include alcohol-related disease, cancer, cardiovascular disease, chronic obstructive pulmonary disease, hypertension, and diabetes mellitus

## 4. Discussion

In the study, we noted that the overall incidence of contracting pneumonia was 1.47-fold higher in the predialysis chronic kidney disease group than in the non-chronic kidney disease group, particularly among the predialysis chronic kidney disease group aged 65-84, who had the highest incidence of contracting pneumonia (45.2 per 1, 000 person-years). Of note, the incidence of contracting pneumonia in patients with predialysis chronic kidney disease appears to be much higher than that in patients with sleep apnea (24.6 vs. 20.9 per 1, 000 person-years), [22] but much lower than that in patients with pulmonary tuberculosis in Taiwan (24.6 *vs*. 51.6 per 1, 000 person-years).[23] From a preventive medicine view, therefore, it is suggested that vaccination for pneumonia might be worth taking into account as an option among patients with predialysis chronic kidney disease, particularly for those with predialysis chronic kidney disease who are aged 65-84 due to their having the highest observed incidence of contracting pneumonia.

After adjusting for co-variables, we noted that patients with predialysis chronic kidney disease demonstrated a 1.52-fold increased hazard of contracting pneumonia, compared with subjects without chronic kidney disease (Table 3). Upon further analysis, patients with predialysis chronic kidney disease alone still had an increased risk of contracting pneumonia (adjusted HR 1.53, Table 4). This finding indicates that patients with predialysis chronic kidney disease alone are at a substantially increased risk for contracting pneumonia even in the absence of any comorbidity. In addition, we noted that if patients were comorbid with predialysis chronic kidney disease and any of the comorbidities studied, the adjusted HR of contracting pneumonia markedly increased to 2.16. This finding indicates that there might be a synergistic effect on the risk of contracting pneumonia between predialysis chronic kidney disease and any of the comorbidities studied.

We can conclude that patients with predialysis chronic kidney disease have a 1.52-fold increased risk of contracting pneumonia as compared to those with non-chronic kidney disease. Thus, even in the absence of any comorbidity, the risk of contracting pneumonia can be said to be high among patients with predialysis chronic kidney disease.
